# Complications Leading to Keratoplasty among Contact Lens Users and LASIK Patients: A 10-Year Cross-Sectional Analysis

**DOI:** 10.1155/2021/5563545

**Published:** 2021-08-13

**Authors:** Sloan W. Rush, Blaze Bulla, Ryan B. Rush

**Affiliations:** ^1^Panhandle Eye Group, 7400 Fleming Ave., Amarillo, TX 79106, USA; ^2^Texas Tech University Health Science Center, 1400 S. Coulter, Amarillo, TX 79106, USA; ^3^Southwest Retina Specialists, 7411 Wallace Blvd., Amarillo, TX 79106, USA

## Abstract

**Purpose:**

To determine the incidence and outcomes in patients who underwent penetrating keratoplasty (PK) resulting from complications related to contact lens (CL) use and laser in situ keratomileusis (LASIK) in a metropolitan area of the United States.

**Methods:**

Population data was obtained from the United States Census Bureau and the Centers for Disease Control. A retrospective, cross-sectional chart review was performed on all patients who underwent keratoplasty in a specific metropolitan geographic area over a ten-year period. The main outcome was best-corrected visual acuity (BCVA) at 2 years in patients who underwent PK secondary to complications related to CL use and LASIK. The secondary outcome was the relative risk of undergoing PK secondary to a complication related to CL use versus LASIK.

**Results:**

The study's geographic area had 46,545 CL users in one or both eyes during any given year and 10,285 patients who underwent LASIK in one or both eyes during the study interval. There were 24 CL users (0.52 per 1,000) and 3 post-LASIK patients (0.29 per 1,000) who underwent PK secondary to complications during the study interval (OR 1.77 [0.53–5.87, 95% CI]; *p*=0.35). BCVA at 2 years was 1.45 [1.0–1.90] logMAR (20/564 Snellen) in the CL using cohort and 0.07 [−1.19–1.33] logMAR (20/23 Snellen) in post-LASIK cohort following PK (*p*=0.04).

**Conclusions:**

Patients who underwent PK secondary to complications related to CL use had worse visual outcomes at 2 years compared to those related to LASIK. Complications leading to PK were rare in both cohorts, but the incidence of undergoing PK secondary to CL use trended higher than LASIK.

## 1. Introduction

Numerous reports have demonstrated safety and efficacy with contact lens (CL) use and laser in situ keratomileusis (LASIK) for correction of refractive error [1–3]. Although uncommon, there still exists significant risk to ocular health in both settings. Complications associated with CL wear include traumatic corneal abrasions, infectious corneal ulcers, acquired limbal stem cell deficiency, and dry eye and other forms of chronic keratoconjunctivitis [4, 5]. CL-associated infiltrates may occur in up to 6% CL users [6] and account for hundreds of thousands of clinic and emergency room visits [7]. Risk factors associated with infection and vision loss from CL use include overnight wear, soft lenses, and hygienic factors related to the use of disinfecting solutions and storage cases [8, 9]. By contrast, morbidities associated with LASIK include dry eye disease, flap irregularities, epithelial downgrowth, central toxic keratopathy, postoperative infection, and corneal ectasia [10–12]. Corneal ectasia in particular remains an important cause of vision loss as it can occur in otherwise healthy eyes with no identifiable risk factors [13]. However, the well-described risk factors associated with ectasia and vision loss from LASIK include thin preoperative pachymetry, anterior corneal topography irregularities, percent tissue thickness alteration, and low residual stromal bed [14, 15].

New technological advances with both CL wear [16] and LASIK [17] have resulted in an improved safety profile but have not eliminated all risks. Most complications occurring with CL wear and LASIK result in temporary and minor vision loss which can be managed medically or with minimally invasive procedures such as phototherapeutic keratectomy (PTK) for scarring [18] or corneal collagen crosslinking (CXL) for ectasia [19, 20]. However, CL use or LASIK can result in complications leading to penetrating keratoplasty (PK) for a variety of reasons. To our knowledge, there are no reports in the literature comparing outcomes of CL use to LASIK as it relates to severe complications leading to PK. In this study, the authors compare the incidence and outcomes in patients who underwent PK resulting from complications related to CL use versus LASIK in a metropolitan area of the United States.

## 2. Methods

The Panhandle Eye Group Institutional Review Board (IORG0009239; IRB00011013) authorized this retrospective case series of patients who underwent PK for complications arising from CL use or LASIK from January 1, 2008, through December 31, 2018, for the statistical area of Amarillo, TX, USA. All components of the study observed the tenets of the Declaration of Helsinki and were executed in accordance with accepted human research requirements and regulations.

### 2.1. Study Population Determinations and Data Collection

The study population for the designated geographic area was determined using published data from the United States Census Bureau [21]. Combined metropolitan and micropolitan statistical areas [22] of the service area that encompassed all adjoining counties of the principal city, Amarillo, TX, were added together. LASIK procedures performed during the study interval were tabulated by obtaining records from all refractive surgery centers in the same statistical population area over the 10-year study period. The average number of contact lens wearers for the study population during the study interval was estimated through published data available from the Centers for Disease Control (CDC) [23, 24]. Medical records were reviewed to identify all subjects undergoing PK during the study interval. The demographic data, clinical features, and visual outcomes were recorded.

### 2.2. Outcomes

The main outcome was best-corrected visual acuity (BCVA) at 2 years (730 ± 60 days) in patients who underwent PK secondary to complications related to CL use and LASIK. The secondary outcome was the relative risk of undergoing PK secondary to a complication related to CL use and LASIK during the study period.

### 2.3. Statistical Analysis

Snellen visual acuity was converted into logMAR for statistical analysis. The JMP 11 software from the SAS Institute (Cary, NC, USA) was used to calculate means and standard deviations. Odds ratios and one-way analysis of the variance was used to compare outcomes and means. The results were considered statistically significant at the alpha <0.05 level.

## 3. Results

The study population for the designated geographic area was 339,744, for which it was estimated that an average of 46,545 people were CL wearers in one or both eyes during any given year of the study period (CDC estimated 13.7% of the study population were CL wearers). A total of 10,285 people underwent LASIK in one or both eyes during the study period. There were 762 patients who underwent keratoplasty (partial or full-thickness) for any reason during the study period in the designated geographic area.

The demographic features and clinical outcomes for patients who underwent PK secondary to complications related to CL use and LASIK are summarized in Table 1. In total, there were 24 patients that underwent PK as a consequence of CL-related complications (0.52 per 1,000), and there were 3 patients who underwent PK as a consequence of complications associated with LASIK (0.29 per 1,000) (OR 1.77 [0.53–5.87, 95% CI]; *p*=0.35).

The mean BCVA improved from a baseline of 2.08 (±0.57) logMAR to 1.45 (±1.10) logMAR in the CL-associated PK group at a 2-year follow-up (*p*=0.02), while the mean BCVA improved from a baseline of 1.06 (±0.22) logMAR to 0.07 (±0.12) logMAR in the LASIK-associated PK group at a 2-year follow-up (*p*=0.002). BCVA, 2 years post-PK, was significantly better in the post-LASIK cohort than that in the CL cohort (*p*=0.04). All three patients in the LASIK-associated PK group achieved vision well enough to receive an unrestricted driver's license (logMAR 0.3 or better) at a 2-year follow-up. Thirteen patients (54.2%) in the CL-associated PK group were legally blind (logMAR 1.0 or worse) at a 2-year follow-up, and 3 patients (12.5%) in the CL-associated PK group had no light perception in the operative eye at a 2-year follow-up.

For the CL-associated PK group, 12 patients (50%) underwent PK for corneal scarring following resolution of an infected corneal ulcer, whereas the other 12 (50%) patients underwent PK as a therapeutic treatment secondary to unresponsiveness to fortified antibiotics. Of the 12 patients who underwent therapeutic PK, 6 (50%) had a full-thickness perforation prior to PK. With regard to the LASIK-associated PK group, all three cases were due to postrefractive ectasia, and there were none due to flap-associated complications or infectious keratitis. There were 29 CL wearers (0.62 per 1,000) and 19 LASIK patients (1.85 per 1,000) who successfully underwent PTK for corneal scarring and CXL for postrefractive ectasia, respectively, to avert PK during the study period.

## 4. Discussion

To our knowledge, this is the first case series to analyze methods of refractive correction directly in a fixed geographic population in regard to complications leading to PK as well as PK outcomes in such patient groups. The incidence of complications leading to PK in CL users and post-LASIK patients was found to be low in our overall study population and not statistically different between groups, although there was a trend towards a higher frequency in CL wearers. However, patients undergoing PK secondary to CL-associated complications had significantly worse visual outcomes at 2 years compared to those with LASIK-associated complications. An alarming number of patients, 40% and 12%, who underwent PK secondary to a CL-associated complication ended up legally blind and with no light perception, respectively. These findings suggest that patient perceptions regarding implied or absolute safety of CL may be misplaced. This study helps underscore the fact that, relative to routine CL wear, refractive surgery is a safe alternative and does not increase the risk for substantial vision loss due to a complication.

The corneal culture results in the CL-associated PK group were positive in 62.5% of our cases for which *Pseudomonas* species was the most common. This is comparable to previous reports [25]. Similar to other studies [26], fungal keratitis cases in our study had the worst visual outcomes. Though not observed in this study, there have been case reports of infectious keratitis associated with LASIK leading to PK [27, 28]. Other investigators have reported that the risk for an infectious corneal ulcer-related event decreases in the setting of treatment with LASIK beyond a 1-year period of contact lens wear [29].

Post-LASIK ectasia requiring intervention has been reported to be as low as 0.033% [10] which is similar to our findings when one considers the incidence of patients who underwent CXL in addition to PK during the study period. Scleral contact lens technology [30], CXL [19, 31], and intracorneal ring segments [32] can provide excellent outcomes and obviate the need for PK. Other LASIK complications besides ectasia include chronic dry eye syndrome, visual aberrations and decentered ablations, diffuse lamellar keratitis, epithelial downgrowth, infectious keratitis, and various flap complications. These types of adverse events have been reported to be as high as 1.3% [33], but our study suggests that they only rarely lead to PK.

Strengths of this study include its analysis of an isolated geographic population to ensure capture of most relevant study cases, its relatively long follow-up period, and its completeness of data. Weaknesses of this study include its retrospective design, the use of logMAR rather than ETDRS letter scoring, reliance on previously published population studies to extrapolate CL use in our study's geographic area, and the relatively small metropolitan size of the study population. An inherent weakness of our study design is that it assumes the number of patients that have either moved away to a different area or have left to receive care from a location outside of the study's metropolitan area is similar to the number patients that have either moved into or sought care inside the study's metropolitan area. Furthermore, it assumes that patients not seeking care are similar to those presenting for care and opting for PK when needed in both CL and LASIK groups.

In conclusion, patients who underwent PK secondary to complications related to CL use had worse visual outcomes at 2 years compared to those related to LASIK. Complications leading to PK were rare in both groups, but the incidence of undergoing PK secondary to CL use trended higher than LASIK. Further research is warranted to validate the findings reported in this study and to determine more factors that will improve safety and further mitigate risk associated with all forms of refractive correction options beyond glasses.

## Figures and Tables

**Table 1:** Complications resulting in penetrating keratoplasty among contact lens users and LASIK patients: demographic features and characteristics.

Group	Age	Gender	Indication for surgery	Culture results	Type of contact lens	Contributing hygienic factors	Preoperative best-corrected visual acuity (Snellen and logMAR)	Postoperative best-corrected visual acuity at 2 years (Snellen and logMAR)	Postoperative complications
*Contact lens-associated complication*, *N* *=* 24 (88.9%)	18	F	Corneal scar after resolved infectious keratitis	*Pseudomonas*	Soft extended wear	No	CF1 (2.0)	20/40 (0.3)	None
	18	F	Corneal scar after resolved infectious keratitis	*Pseudomonas*	Soft extended wear	Yes	CF3 (1.8)	20/20 (0)	Graft rejection
	24	F	Unresponsive infectious ulcer without perforation	*Metarrhizium*	Soft daily disposable	No	LP (2.7)	20/100 (0.7)	None
	25	M	Unresponsive infectious ulcer without perforation	*Fusarium*	Soft extended wear	Yes	LP (2.7)	NLP (3.0)	Phthisis bulbi with evisceration
	26	M	Corneal scar after resolved infectious keratitis with perforation	Negative	Soft extended wear	Yes	HM1 (2.4)	HM1 (2.4)	None
	30	M	Corneal scar after resolved infectious keratitis	*Pseudomonas*	Soft extended wear	Yes	20/200 (1.0)	20/30 (0.2)	None
	32	M	Corneal scar after resolved infectious keratitis	Negative	Soft extended wear	Yes	HM1 (2.4)	LP (2.7)	Glaucoma
	33	F	Corneal scar after resolved infectious keratitis with perforation	Negative	Soft extended wear	Yes	HM3 (2.2)	HM1 (2.4)	None
	34	F	Corneal scar after resolved infectious keratitis	Negative	Soft daily disposable	No	20/300 (1.2)	20/20 (0)	None
	35	M	Unresponsive infectious ulcer with perforation	Negative	Soft extended wear	Yes	HM1 (2.4)	20/150 (0.88)	Graft rejection
	38	M	Unresponsive infectious ulcer with perforation	*Pseudomonas*	Soft extended wear	Yes	LP (2.7)	CF1 (2.0)	Endophthalmitis
	42	F	Unresponsive infectious ulcer without perforation	*Aspergillus*	Soft extended wear	No	LP (2.7)	NLP (3.0)	Phthisis bulbi
	44	F	Corneal scar after resolved infectious keratitis with perforation	Atypical acid-fast bacilli	Soft extended wear	Yes	HM3 (2.2)	HM1 (2.4)	Glaucoma atrophia bulbi
	45	F	Corneal scar after resolved infectious keratitis	Negative	Soft extended wear	Yes	CF3 (1.8)	20/70 (0.54)	Glaucoma
	53	M	Unresponsive infectious ulcer with perforation	*Haemophilus*	Soft extended wear	Yes	LP (2.7)	CF1 (2.0)	Recurrent corneal ulcer
	53	M	Unresponsive infectious ulcer without perforation	*Candida*	Soft extended wear	No	HM3 (2.2)	20/250 (1.1)	None
	55	F	Unresponsive infectious ulcer with perforation	*Staphylococcus aureus*	Soft extended wear	Yes	HM3 (2.2)	HM1 (2.4)	None
	56	F	Unresponsive infectious ulcer with perforation	Methicillin-resistant *Staphylococcus aureus*	Soft extended wear	Yes	CF3 (1.8)	HM (2.4)	Glaucoma
	57	F	Unresponsive infectious ulcer without perforation	Negative	Soft extended wear	No	LP (2.7)	NLP (3.0)	None
	59	F	Unresponsive infectious ulcer with perforation	*Fusarium*	Soft daily disposable	No	HM3 (2.2)	20/25 (0.1)	None
	59	F	Corneal scar after resolved infectious keratitis	*Staphylococcus aureus*	Soft daily disposable	Yes	20/200 (1.0)	CF1 (2.0)	Graft rejection
	61	F	Corneal scar after resolved infectious keratitis	*Fusarium*	Soft extended wear	No	20/400 (1.3)	20/70 (0.54)	None
	64	F	Unresponsive infectious ulcer without perforation	Negative	Soft extended wear	No	HM1 (2.4)	20/60 (0.48)	None
	74	M	Corneal scar after resolved infectious keratitis	Negative	Soft extended wear	No	20/400 (1.3)	20/40 (0.3)	None

*LASIK-associated complication*, *N* *=* 3 (11.1*%)*	41	F	Postrefractive ectasia	None	None	No	20/200 (1.0)	20/20 (0)	None
	45	M	Postrefractive ectasia	None	None	Yes (eye rubbing)	20/400 (1.3)	20/30 (0.2)	None
	52	M	Postrefractive ectasia	None	None	No	20/150 (0.88)	20/20 (0)	None

LP, light perception; HM, hand motion; CF, count fingers; NLP, no light perception.

## Data Availability

The datasets used and/or analyzed during the current study are available from the corresponding author upon reasonable request.
